# Gypenoside XVII, an Active Ingredient from Gynostemma Pentaphyllum, Inhibits C3aR-Associated Synaptic Pruning in Stressed Mice

**DOI:** 10.3390/nu14122418

**Published:** 2022-06-10

**Authors:** Man-Man Zhang, Guo-Ming Huo, Jie Cheng, Qiu-Ping Zhang, Na-Zhi Li, Min-Xia Guo, Qing Liu, Guang-Hui Xu, Ji-Xiao Zhu, Cheng-Fu Li, Feng Zhou, Li-Tao Yi

**Affiliations:** 1 Department of Chemical and Pharmaceutical Engineering, Huaqiao University, Xiamen 361021, China; 20013087011@stu.hqu.edu.cn (M.-M.Z.); chengj@hqu.edu.cn (J.C.); liuq@hqu.edu.cn (Q.L.); 2 School of Food Science, Nanjing Xiaozhuang University, Nanjing 211171, China; cnhuogm@njxzc.edu.cn; 3 Xiamen Hospital of Traditional Chinese Medicine, Xiamen 361009, China; 15639775331@163.com (Q.-P.Z.); lichengfu103@126.com (C.-F.L.); 4 Research Center of Natural Resources of Chinese Medicinal Materials and Ethnic Medicine, Jiangxi University of Traditional Chinese Medicine, Nanchang 330004, China; l418698667@163.com (N.-Z.L.); guo144229@163.com (M.-X.G.); zhujx81@sina.com (J.-X.Z.); 5 Xiamen Medicine Research Institute, Xiamen 361008, China; xghcxm@163.com

**Keywords:** Gypenoside XVII, antidepressant, complement C3, synaptic pruning, microglia

## Abstract

Gynostemma pentaphyllum is a herbal medicine widely used in Asian countries, and its saponin extracts have been shown to possess potent anti-inflammatory effects. Gypenoside XVII, an active ingredient isolated from Gynostemma pentaphyllum, has been found to alleviate the inflammation induced by LPS in the BV2 microglia, according to our preliminary study. This study aims to evaluate whether Gypenoside XVII could attenuate depression-like symptoms in vivo and tries to demonstrate the involvement of the complement regulation in its antidepressant-like effect. The results showed that Gypenoside XVII significantly attenuated depression-like behaviors in the forced swimming test, tail suspension test and sucrose preference test. It also alleviated the acute stress-induced hyperactivity of serum corticosterone levels. Additionally, Gypenoside XVII significantly inhibited the activation of microglia and the expression of C3 in mice exposed to chronic unpredictable mild stress (CUMS). Meanwhile, the activation of C3aR/STAT3 signaling and the expression of proinflammatory cytokines was reversed by Gypenoside XVII. Moreover, CUMS induced excessive synaptic pruning by activating microglia, while Gypenoside XVII restored it in the prefrontal cortex. Our data demonstrated that Gypenoside XVII, the active ingredient of Gynostemma pentaphyllum, produced the antidepressant-like effects in mice, which was mediated by the inhibition of complement C3/C3aR/STAT3/cytokine signaling in the prefrontal cortex.

## 1. Introduction

Depression is a common mental disorder and one of the most important public health problems in the world. Increasing evidence shows a close connection between inflammation and depression [1,2]. The key to the influence of neuroinflammation on depression lies in the imbalance of the control and release of proinflammatory and anti-inflammatory cytokines. The neuroinflammatory response is mainly mediated by glial cells, especially microglia, which are the natural resident macrophage in the central nervous system.

*Gynostemma pentaphyllum* is a perennial plant that is widely distributed in the subtropical and northern subtropical regions [3]. It has been used as a herbal medicine for thousands of years [4]. Gypenosides is a total saponin extract isolated from *Gynostemma pentaphyllum*, which includes more than 100 Gypenosides [5,6,7]. A previous study has shown that Gypenosides could alleviate insulin resistance and decrease the risk of being overweight [8]. In addition, our previous studies found that Gypenosides could reverse depression-like symptoms in various depression models [9,10]. Moreover, several other reports and our previous research have demonstrated that the anti-inflammatory activity of Gypenosides mediated the therapeutic roles in memory and cognitive impairments [11,12,13]. However, little is known about which active ingredient mediates the antidepressant-like effects of Gypenosides.

The response state of microglia to the pathological response has been called microglia activation, and any pathological event in the brain will lead to the activation of microglia. In a normal brain, microglia are considered “resting”. However, once microglia are activated, they can remove damaged cells and dysfunctional synapses [14]. This process is called “synaptic pruning”. In some cases, the entire dendritic tree will be eliminated during the synaptic pruning. Due to the release of many substances, including cytokines, chemokines and growth factors, microglia can strongly influence the pathological results or responses to stressors [15]. Therefore, microglia-mediated neuroinflammation is considered a possible cause of the occurrence or deterioration of depression. On the contrary, inhibiting the excessive activation of microglia and inhibiting neuroinflammation may be an effective method for treating depression.

The complement system is an important part of the innate immune system, which can enhance or supplement the ability of antibodies and macrophages to clear damaged cells and microorganisms and to promote the process of inflammatory response. It is an effector system and effector amplification system with a crucial biological significance in the body. Complement is widely involved in physiological processes, such as the development of the nervous system, the elimination of synapses, and the maturation of neural networks. Excessive activation of the complement system and the increased levels of complement C3 protein in the central nervous system can lead to neuroinflammatory reactions and neuronal damage [16]. Recent studies have shown that the inhibition of complement activation has a neuroprotective effect when mental disease occurs [17,18], which provides a new method for preventing and treating depression.

In our preliminary study, the active ingredient Gypenoside XVII was screened from the total Gypenosides of *Gynostemma pentaphyllum*, which was based on the Traditional Chinese Medicine Systems Pharmacology (TCMSP) database. We found that Gypenoside XVII could alleviate the inflammation induced by LPS in the BV2 microglia. However, it remains unclear whether Gypenoside XVII could exert antidepressant-like responses in vivo, and whether Gypenoside XVII attenuates depression via regulating the complement system. Therefore, the present study aimed to investigate the effects and elucidate the underlying complement-mediated mechanism of Gypenoside XVII in various depression-like models.

## 2. Materials and Methods

### 2.1. Animals

Male C57BL/6 mice (26 ± 2 g; 8 weeks old) were purchased from the Shanghai Slac Animal Center, Shanghai, China. The animals were housed five per cage (320 × 180 × 160 mm) under a normal 12 h/12 h light to dark schedule, with the lights on at 07:00 a.m. The animals were allowed to adjust to the housing conditions before the experiments began. An ambient temperature and relative humidity were maintained at 22 ± 2 °C and at 55 ± 5 %, respectively. During the entire experiment, the animals had access to food and water unless otherwise specified. All the procedures were approved by Huaqiao University (A2020002) and performed in accordance with the published guidelines of the China Council on Animal Care.

### 2.2. Chemicals and Reagents

Gypenoside XVII, of a large amount, was extracted from *Gynostemma pentaphyllum* and provided by Abmole (Houston, TX, USA). DAPI and primary β-actin antibody were purchased from Sigma (St. Louis, MO, USA). The antibodies for C3 (ab97462), PSD95 (ab18258), vGluT1 (ab227805) and Iba1 (ab5076) were purchased from Abcam (Cambridge, MA, USA). The antibody for C3aR (sc-133172) was purchased from Santa Cruz (Freemont, CA, USA).

### 2.3. Drug Administration

Firstly, in order to investigate the putative antidepressant-like effects of Gypenoside XVII and to obtain an optimal dose for the long-term experiment, the mice were randomized into six groups: vehicle, fluoxetine (20 mg/kg) and Gypenoside XVII (1, 2.5, 5 and 10 mg/kg). The number of mice in each group was 8. The drugs were orally administrated only once. The behavioral tests, including the open-field test, forced swimming test and tail suspension test, were performed after the drug administration. After the behavioral tests, mice were sacrificed, and the blood was collected for serum separation.

The minimum effective dose was selected for the long-term experiment according to the results from acute administration. In order to elucidate the mechanism of Gypenoside XVII, sixty mice were randomly divided into six groups (*n* = 10): the Normal/CUMS-vehicle groups that received the vehicle (0.9% saline containing 0.3% carboxymethyl cellulose), the Normal-Gypenoside XVII/CUMS-Gypenoside XVII groups that received 2.5 mg/kg of Gypenoside XVII, the CUMS-Gypenosides groups that received 100 mg/kg of Gypenosides, and the CUMS-fluoxetine groups that received 20 mg/kg of fluoxetine.

Gypenosides, Gypenoside XVII and fluoxetine were dissolved in 0.9% saline containing 0.3% carboxymethyl cellulose and were administered by oral gavage once daily for 4 continuous weeks. The dose of fluoxetine was selected based on a previous report [19].

### 2.4. Open-Field Test

The mice were placed in the center of the experimental box (40 cm × 40 cm × 30 cm divided into 25 equal squares (8 cm × 8 cm)), and the following two indices were measured within 5 min of each mouse: firstly, the mouse’s two front paws were 1 cm or more away from the bottom of the box or clinging to the box wall, and the two front paws were recorded as rearing. Secondly, the number of horizontal crossing grids (at least two paws entering a grid was recorded as one grid.

### 2.5. Tail Suspension Test

The mouse’s tail was fixed with tube clamps, and the mouse’s head was suspended 10 cm from the bottom of the box. The mice were suspended for 6 min. The experimental sessions were recorded by video. The immobility time of each mouse was counted from the last 4 min video. The immobility was considered a state where mice stopped struggling.

### 2.6. Forced Swimming Test

The mice were placed in a cylindrical glass container filled with water at the height of 15 cm, and the temperature was maintained at 25 °C. The mice were forced to swim in the container for 6 min. The experimental sessions were recorded by video. The immobility time of each mouse was counted from the last 4 min video. The immobility was considered a state where the head of the mouse was kept above the water’s surface and the limbs were floating. 

### 2.7. Corticosterone Measurement

After the forced swimming test of acute administration, the blood was collected from the mice. The serum was separated by centrifuging at 3000× *g* for 10 min. Then, the serum was diluted and the corticosterone levels were measured by the ELISA method according to the manufacturer’s instructions (Enzo Life Sciences, Farmingdale, NY, USA).

### 2.8. Chronic Unpredictable Mild Stress (CUMS)

All mice except for the normal mice were continuously and randomly given the following stressors every day: water and food deprivation, circadian lighting, empty water bottle, dirty cage, reduced space, 45° inclined cage, light/dark interval lighting, strobe lighting and noise. Normal mice were housed in individually separated rooms and were not disturbed by the stressors. The CUMS procedure lasted for 8 weeks, and the sucrose preference was performed as a sign of successful modeling every week.

### 2.9. Sucrose Preference Test

First, the mice were trained to drink sucrose solution (1%) for 24 h. Then, both the sucrose solution and water were placed into the cage for drinking for another 24 h. During this period, the positions of the two bottles should be changed every 12 h. After the adaptive training, the mice were deprived of water and food for 12 h, and the formal experiment started in a separated cage: the mice were exposed to both the sucrose solution and water. After 24 h, the sucrose solution and water consumption were recorded, and the sucrose preference was calculated.

### 2.10. Western Blotting

The prefrontal cortex was lysed with protein lysis buffer. Then, the solution was centrifuged, and the supernatant was collected. The protein concentration was detected by the BCA method. The concentrations of the samples were adjusted to be equal. Then, 30 μg of protein was loaded in the SDS-PAGE gel for electrophoresis. Next, the proteins were transferred from the gel to the PVDF membranes, followed by blocking with 5% BSA at room temperature for 1 h. Subsequently, primary antibodies were incubated at 4 °C overnight on a shaker. The secondary antibody was added and incubated at room temperature for 1 h on a shaker. Finally, the ECL luminescent solution was added to the membrane, followed by photographing. β-actin was used as the internal reference in the present study.

### 2.11. Real-Time PCR

The total RNA from the prefrontal cortex of mice was extracted by the Trizol reagent, and the total RNA was reverse transcribed into cDNA and used as a template according to the instructions of the reverse transcription kit. According to the reaction condition of the fluorescence quantitative PCR kit, the Ct value was tested in the Bio-Rad CFX96 Touch PCR machine. The following process was used: pre-denaturation at 94 °C for 2 min, denaturation at 94 °C for 15 s, and annealing/extension at 60 °C for 30 s, 40 cycles. The 2^−^^ΔΔCt^ method was used to calculate the expression of the proinflammatory cytokines. 

### 2.12. Brain Extraction and Immunofluorescence

Mice were anesthetized with pentobarbital sodium and then perfusion with PBS and 4% paraformaldehyde, respectively. The whole brain was carefully dissected and fixed at 4% paraformaldehyde. Further, 24 h later, the brains were subsequently incubated in an increasing gradient of sucrose solution from 10% to 30%, followed by being embedded with OCT in a 30% sucrose solution. Then, 15-μm-thick sections were cut according to the previous publication [20] and post-fixed at 4% paraformaldehyde again. Thereafter, the sections received an antigen retrieval and were blocked with a blocking buffer for 1h. The sections were incubated with anti-iba1 (1:200), anti-C3 (1:150), anti-C3aR (1:150), anti-PSD95 (1:100), or anti-vGluT1 (1:100) overnight. Then, the sections were washed with TBST and then incubated with relative fluorescence antibodies for 3 h. Then, DAPI (Sigma; 1:5000) was added after washing by TBST. Finally, the signals were detected and were observed under a confocal microscope (Leica TCS SP8). 

### 2.13. Statistical Analyses

All data are expressed as the mean ± SEM. Generally, suppose we want the SEM less than 10% of the mean. In that case, we need more than 8 animals in the behavioral test, more than 6 samples in the ELISA/PCR assay, and more than 4 samples for the Western blot/immunofluorescence measurement. The data were analyzed using a one-way or two-way ANOVA, followed by a Tukey’s test. A value of *p* < 0.05 was considered to be statistically significant for the analysis.

## 3. Results

### 3.1. Gypenoside XVII Exhibited the Antidepressant-Like Effects in Mice

**Figure 1:**
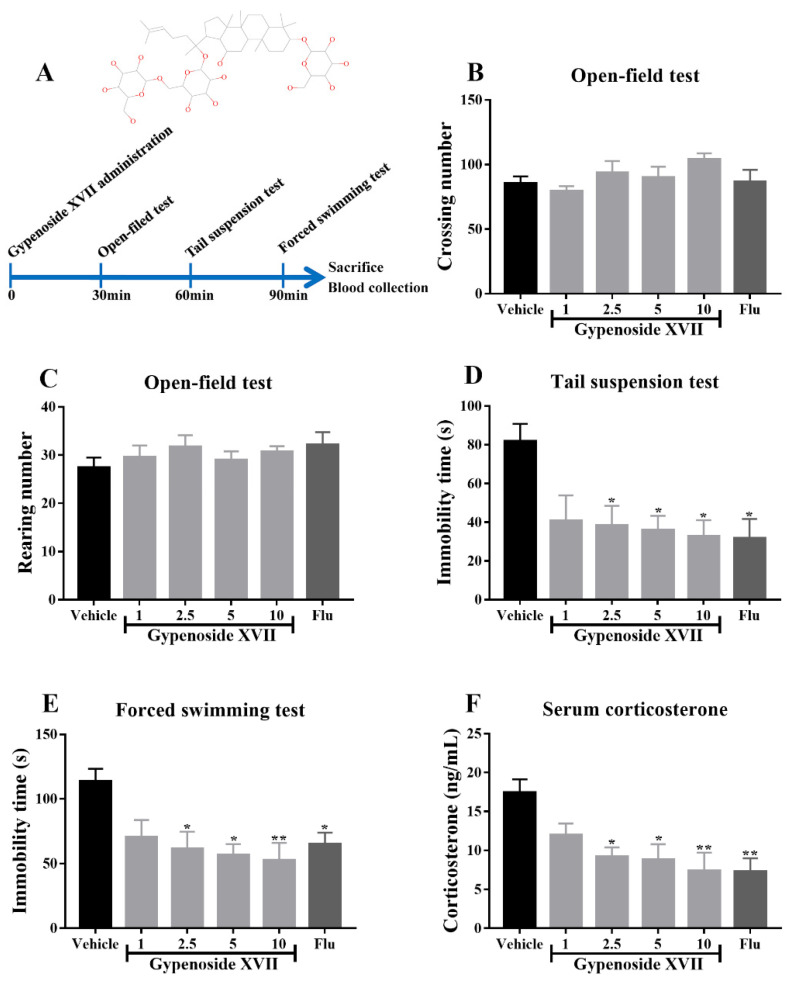
Acute administration with Gypenoside XVII decreased the immobility time but did not affect locomotor activity in the behavioral tests (*n* = 8). The structure of Gypenoside XVII and the experimental timeline (**A**). Open-field test (**B**,**C**). Tail suspension test (**D**). Forced swimming test (**E**). Serum corticosterone levels (**F**). * *p* < 0.05 and ** *p* < 0.01 versus the vehicle group.

According to Figure 1A,B, an acute administration with Gypenoside XVII did not affect the crossing number or rearing number in the open-field test. Meanwhile, Gypenoside XVII significantly decreased the immobility time in the tail suspension test and the forced swimming test (Figure 1C,D). The best therapeutic effects were observed at 10 mg/kg of Gypenoside. In addition, an acute Gypenoside XVII administration dramatically decreased the serum corticosterone levels after the despair behavioral tests (Figure 1E). Taken together, these observations indicate the antidepressant-like properties of Gypenoside XVII.

### 3.2. Gypenoside XVII Attenuated the Depressive-Like Symptoms in CUMS Mice

**Figure 2:**
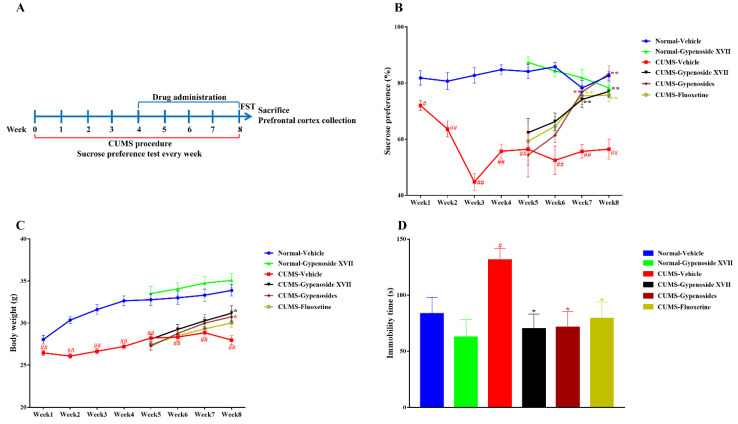
Gypenoside XVII reversed depression-like symptoms in depression-like mice (*n* = 10). Diagram for the experimental protocol (**A**). Sucrose preference during an 8-week CUMS procedure (**B**). Body weight during an 8-week CUMS procedure (**C**). Immobility time at the end of CUMS procedure (**D**). # *p* < 0.05 and ## *p* < 0.01 versus the Normal-vehicle group. * *p* < 0.05 and ** *p* < 0.01 versus the CUMS-vehicle group.

The sucrose preference was measured every week during the 8-week CUMS procedure. CUMS induced a significant reduction in the sucrose preference after a 1-week exposure, suggesting the anhedonia of mice (Figure 2A). This anhedonia symptom lasted in the whole experiment. The normal and CUMS mice were randomly divided into several groups, which were treated with the vehicle, Gypenoside XVII, Gypenosides or fluoxetine. The therapeutic effects appeared three weeks after the drug administration and were enhanced after administration for 4 weeks. Meanwhile, the body weight was also recorded every week. The results showed that CUMS inhibited the body weight gain one week later (Figure 2B). Gypenoside XVII and Gypenosides restored the body weight in the eighth week when the drugs were administrated for 4 weeks. In addition, a forced swimming test was performed immediately after the sucrose preference test. The data indicated that CUMS induced the elevation of the immobility time of the mice, which suggested a despair symptom in animals. On the contrary, all drugs, including Gypenoside XVII, Gypenosides and fluoxetine, markedly decreased the immobility time in the CUMS mice (Figure 2C).

### 3.3. Gypenoside XVII Inhibited Microglia Proliferation and Decreased C3 Expression in the Prefrontal Cortex

**Figure 3:**
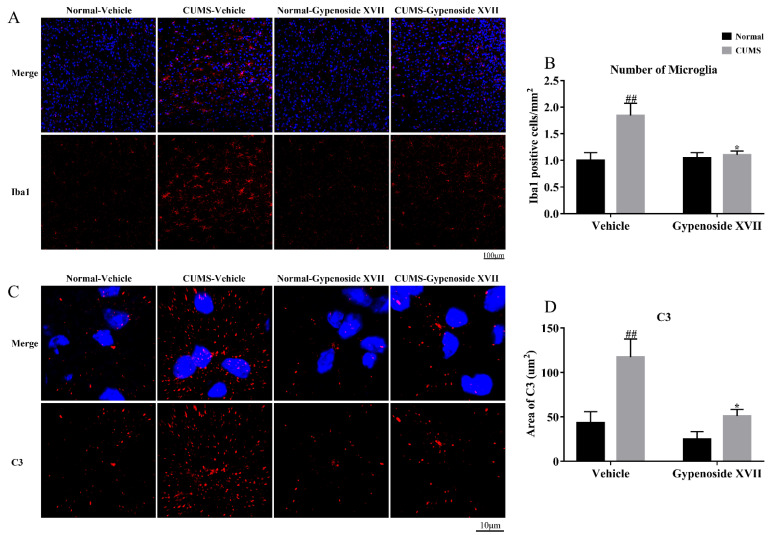
The number of microglia was decreased and the levels of C3 were decreased by Gypenoside XVII in the prefrontal cortex (*n* = 4). The number of microglia (**A**,**B**) and the levels of C3 (**C**,**D**) were detected by immunofluorescence. ## *p* < 0.01 versus the Normal-vehicle group. * *p* < 0.05 versus the CUMS-vehicle group.

According to the results displayed in Figure 3, compared with the Normal-vehicle group, CUMS mice showed a significant increase in the number of Iba1 positive cells, indicating the activation of microglia. After the administration with Gypenoside XVII for four weeks, compared with the CUMS-vehicle group, the number of Iba1 positive cells was decreased. In agreement with the change of Iba1, the levels of C3 were also upregulated in response to the CUMS procedure. This action induced by CUMS was reversed by the Gypenoside XVII administration.

### 3.4. Gypenoside XVII Inhibited C3aR/STAT3/Cytokine Signaling Pathway in CUMS Mice

**Figure 4:**
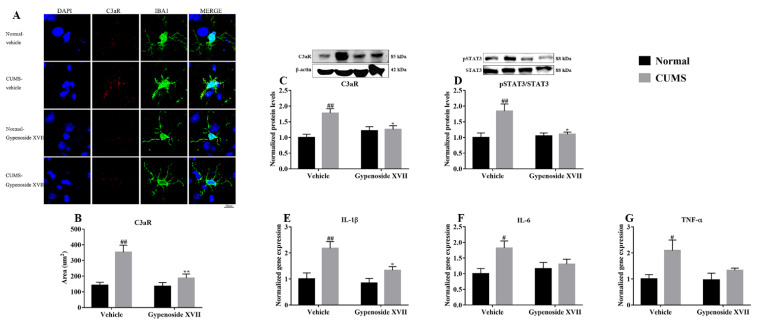
Gypenoside XVII inhibits C3aR-STAT3 cytokines signaling in the prefrontal cortex (*n* = 4–6). C3aR levels were detected by both immunofluorescence (**A**,**B**) and Western blot (**C**). pSTAT3/STAT3 was detected by Western blot (**D**). The mRNA expression of IL-1β (**E**), IL-6 (**F**) and TNF-α (**G**) was detected by PCR. # *p* < 0.05 and ## *p* < 0.01 versus the Normal-vehicle group. * *p* < 0.05 and ** *p* < 0.01 versus the CUMS-vehicle group.

The complement C3-mediated antidepressant-like mechanism of Gypenoside XVII was determined by investigating the C3 receptor, C3aR and its downstream effector STAT3 as well as the target genes, including IL-1β, IL-6 and TNF-α. The immunofluorescence analysis showed that the CUMS treatment significantly enhanced the levels of C3aR in microglia, while Gypenoside XVII could significantly inhibit the expression in microglia (Figure 4A,B). To further validate the change of C3aR in the prefrontal cortex, we performed a Western blot analysis, and the consistent results were obtained, as shown in Figure 4C. Likewise, STAT3, the downstream protein of C3aR, was also activated by CUMS in the prefrontal cortex. The administration of Gypenoside XVII induced the downregulation of pSTAT3 (Figure 4D). Furthermore, the increase in proinflammatory cytokines that were induced by the C3aR/STAT3 signaling in the CUMS mice was also observed by a PCR assay (Figure 4E–G). However, Gypenoside XVII could partly decrease the expression of the proinflammatory cytokines in the prefrontal cortex. These results suggest that Gypenoside XVII might possess potential anti-inflammatory activity via inhibiting the C3aR/STAT3 signaling and suppressing cytokine release.

### 3.5. Gypenoside XVII Inhibited Microglia-Mediated Synaptic Pruning in CUMS Mice

**Figure 5:**
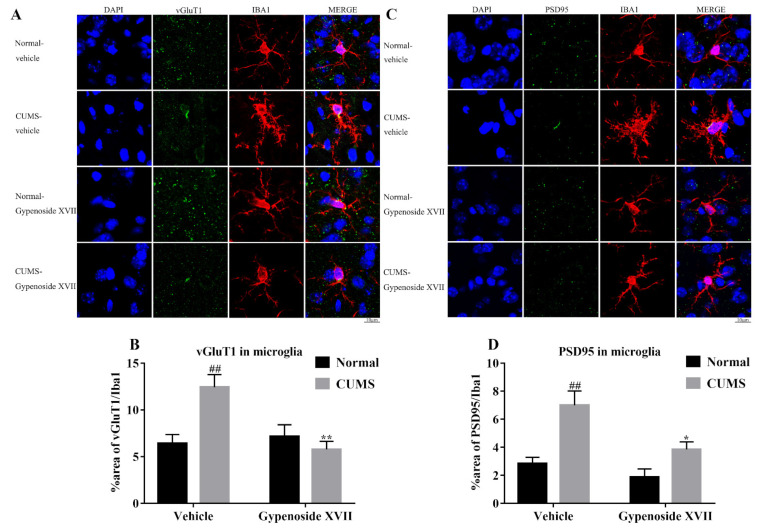
Gypenoside XVII prevented synaptic pruning in the prefrontal cortex (*n* = 4). Presynaptic protein vGluT1 in microglia (**A**,**B**). Postsynaptic protein PSD95 in microglia (**C**,**D**). ## *p* < 0.01 versus the Normal-vehicle group. * *p* < 0.05 and ** *p* < 0.01versus the CUMS-vehicle group.

The role of C3-mediated synaptic pruning in neuroinflammation is well documented; therefore, we tried to investigate whether C3-mediated synaptic pruning was also induced in depression and if it was prevented by Gypenoside XVII. To address this, we stained both the microglial marker, Iba1 and the presynaptic protein marker, vGluT1, in the prefrontal cortex (Figure 5A,B). The results showed that the levels of vGluT1 within the microglia were significantly increased in the CUMS group compared to that of the Normal group. The treatment with Gypenoside XVII induced a remarkable reduction of vGluT1 within the microglia. Furthermore, the postsynaptic protein marker, PSD95, was also stained with the microglial marker, Iba1, in accordance with the results of vGluT1, where CUMS triggered the levels of PSD95 in microglia, while Gypenoside showed the protective activity against the detrimental effects induced by CUMS. Finally, our results revealed that Gypenoside could inhibit C3-induced excessive synaptic pruning in the prefrontal cortex of CUMS mice, which might mediate the potential antidepressant-like effects of Gypenoside.

## 4. Discussion

Bioactive dietary food components have been used in dietary supplements to prevent various mental diseases, including anxiety and depression [21,22]. *Gynostemma pentaphyllum* is a well-known medicinal and food homologous product. Gypenosides are the main active components from *Gynostemma pentaphyllum*, with diverse biological activities. In our preliminary study, Gypenoside XVII, the active ingredient of Gypenosides, was isolated and screened in LPS-induced BV2 microglia. The present study first evaluated its antidepressant-like effects after acute administration. The results indicated that Gypenoside XVII shortened the immobility time in the forced swimming and tail suspension tests. Meanwhile, Gypenoside XVII did not affect the crossing and rearing numbers in the open-field tests. On the other hand, the hyperactivity of the hypothalamic-pituitary-adrenal (HPA) axis is one of the common neurobiological abnormalities in the serum of patients with depression [23]; the biochemical analysis provided evidence that Gypenoside XVII decreased the levels of corticosterone, the core hormone of the HPA axis. These observations above, for the first time, clearly demonstrate the antidepressant-like effects of Gypenoside XVII.

Anhedonia is the core symptom of depression, therefore, the sucrose preference test was firstly used for the evaluation. Our findings demonstrated that Gypenosides, Gypenoside XVII and fluoxetine administration gradually increased the sucrose preference of the CUMS-induced mice to normal levels. In detail, Gypenoside XVII tended to increase the sucrose preference during the first two weeks, although the increase did not reach significance. It significantly reversed the reduction in the sucrose preference beginning at 3 weeks of administration as compared with the CUMS-vehicle animals. At the last detecting point of week 4, Gypenoside XVII still exhibited an increase in the sucrose preference test to that in the CUMS mice. Another prominent symptom of depression is despair. This symptom in the behavioral test means that depressed mice give up struggling with the stress or environment and exhibit a longer immobility time. The results indicated that Gypenoside XVII also decreased the immobility time in the CUMS-induced mice, such as those receiving Gypenosides or fluoxetine. Collectively, these behavioral observations demonstrate that Gypenoside XVII exerts an antidepressant-like effect in mice and could be potentially developed into a novel antidepressant.

Microglia constantly observe the brain for harmful signals, such as pathogens, damages and protein aggregates through their long and rapid movement process [24]. Once microglia detect these signals, they will be activated, that is, they will produce reactive oxygen species, release chemokines and proinflammatory cytokines, upregulate phagocytosis and move to the signals. However, if the harmful signals persist, microglia may be activated for a long time, leading to excessive synaptic pruning and neuronal damage [25]. In the present study, CUMS induced the activation and proliferation of microglia in the prefrontal cortex. On the contrary, the Gypenoside XVII administration for 4 weeks significantly attenuated microglial activation and decreased the number of Iba-1-labeled microglia, which was consistent with our previous study, showing that the total Gypenosides decreased the number of microglia in the CUMS mice.

Recently, a growing number of studies have found that complement-related signaling pathways play an important role in neuropsychiatric diseases, including Alzheimer’s disease and depression [26,27]. It was found that stress caused an abnormal activation of complement-mediated synaptic pruning in microglia [28], which provided new ideas for the treatment of depression in the aspect of complement intervention. Complement C3 is a key protein of the complement cascade, and its multiple molecular binding sites are the key to its function. Complement C3 binds to its responding receptors and plays an important role in the pathological mechanisms of immune defense, inflammation and neuropsychiatric diseases. It has been shown that the role of complement C3 is closely related to microglial activation [29], as its receptor C3aR is a specific pattern recognition receptor for microglia [18]. According to these findings, our study showed that the levels of C3 and C3aR were significantly upregulated in response to chronic stress. While Gypenoside XVII significantly downregulated the levels of C3 and C3aR in the prefrontal cortex, suggesting that the inhibition of the complement system is involved in the antidepressant-like effects of Gypenoside XVII.

Complement-induced cleavage products, such as C3a, are known to trigger neuroinflammation and activation of microglia expressing C3aR, which leads to the induction of chemotaxis, including proinflammatory cytokine production [30]. Generally, C3aR, a G-protein-coupled anaphylatoxin receptor, activates its downstream STAT3. STAT3 plays a crucial role in the regulation of inflammation and immunity. A previous study has shown that microglia knockout STAT3 rescued the depression-like symptoms in mice [31]. Moreover, the modulation of the STAT3 pathway mediates the effects of antidepressants, such as fluoxetine [32]. Our study showed that CUMS caused the significant upregulation of C3aR-STAT3 signaling and proinflammatory cytokines, including IL-1β, IL-6 and TNF-α in the prefrontal cortex, confirming the regulatory property of the C3aR-STAT3 signaling pathway on proinflammatory cytokines and its involvement in the pathophysiology of depression. The proinflammatory factors secreted by activated microglia cause the activation of a larger range of microglia and astrocytes, which repeatedly causes chronic inflammation in the brain for a long time. This may be the mechanism that maintains the continuous activation of microglia. In contrast, Gypenoside XVII potentially reversed the CUMS-induced overexpression in proinflammatory cytokines by restoring C3aR-STAT3 signaling.

The accumulating previous studies have indicated that the binding of the C3aR receptors to C3a can label active synapses [33], and microglia can recognize the labeled synapses and eliminate them. This so-called synaptic pruning is a natural process that is a benefit to maintaining more efficient brain function between early childhood and the onset of puberty. However, excessive synaptic pruning is associated with a synaptic loss in several pathological conditions [34]. This means that it will be detrimental if the function of synaptic pruning is hyperactivated in the adult brain. In the present study, we confirmed that the active component Gypenoside XVII could inhibit complement C3/C3aR-mediated synaptic pruning in microglia by CUMS. This result is in agreement with previous observations that treatment with a C3aR antagonist inhibited inflammation in CUMS-induced depression [28].

**Figure 6:**
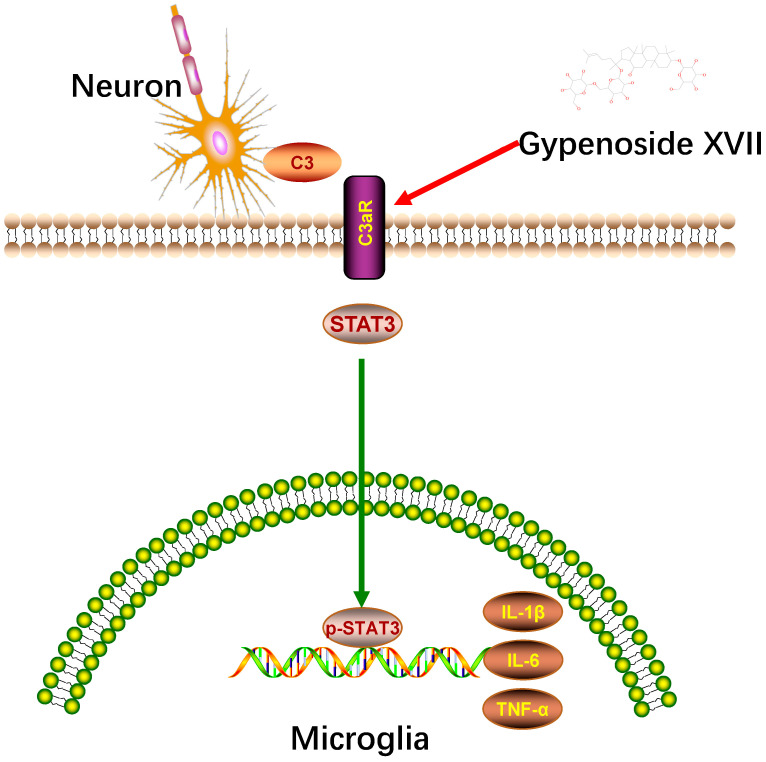
Schematic diagram of the antidepressant-like Gypenoside XVII in CUMS-induced mice. Chronic stress activates microglia and enhances C3 release. C3 targets neuron and recruit microglia to eliminate targeted synapses. Meanwhile, C3 binds with C3aR to activate STAT3. Subsequently, STAT3 induces the activation of the pro-inflammatory cytokine to aggravate the inflammatory response.

In conclusion, our present study verified that Gypenoside XVII is the active ingredient of Gypenosides for its antidepressant-like effects. The results also indicated that Gypenoside XVII from Gypenosides could regulate the complement system by attenuating the changes in the C3/C3aR/STAT3 signaling pathway and thus inhibiting synaptic pruning in stress-induced depression (Figure 6). Therefore, the present study reveals the components basis of Gypenosides and provides fundamental evidence for its further development as a valuable complement regulatory reagent.

## Data Availability

The data in this study are available on reasonable request from the corresponding author.
